# The Potential of Transgenic Hybrid Aspen Plants with a Recombinant *Lac* Gene from the Fungus *Trametes hirsuta* to Degrade Trichlorophenol

**DOI:** 10.3390/genes16030298

**Published:** 2025-02-28

**Authors:** Elena O. Vidyagina, Natalia M. Subbotina, Eugenia N. Belova, Yulia A. Kovalitskaya, Vyacheslav A. Evdokimov, Vladimir A. Belyi, Alexey P. Kochetov, Alexey K. Surin, Konstantin V. Krutovsky, Konstantin A. Shestibratov

**Affiliations:** 1 Branch of the Shemyakin-Ovchinnikov Institute of Bioorganic Chemistry, Russian Academy of Sciences, Prospekt Nauki 6, 142290 Pushchino, Russia; vidjagina@mail.ru (E.O.V.); natysubbotina@mail.ru (N.M.S.); zhenitchka@yandex.ru (E.N.B.); alan@vega.protres.ru (A.K.S.); 2 Institute of Cell Biophysics, Russian Academy of Sciences, Institutskaya 3, 142290 Pushchino, Russia; kovalitskaya@inbox.ru (Y.A.K.); evdokimov@mail.ru (V.A.E.); 3 Institute of Chemistry, Komi Science Centre, Urals Branch of the Russian Academy of Sciences, Republic of Komi, Pervomaiskaya Str. 48, 167000 Syktyvkar, Russia; skeyling@yandex.ru; 4 Pushchino State Institute of Natural Sciences, Prospekt Nauki 3, 142290 Pushchino, Russia; 5 State Research Center for Applied Microbiology and Biotechnology, 142279 Obolensk, Russia; 6 Institute of Protein Research, Russian Academy of Sciences, 142290 Pushchino, Russia; 7 Department of Forest Genetics and Forest Tree Breeding, Georg-August University of Göttingen, 37077 Göttingen, Germany; 8 Center for Integrated Breeding Research, George-August University of Göttingen, 37075 Göttingen, Germany; 9 Laboratory of Forest Genomics, Genome Research and Education Center, Institute of Fundamental Biology and Biotechnology, Siberian Federal University, 660041 Krasnoyarsk, Russia; 10 Department of Genomics and Bioinformatics, Institute of Fundamental Biology and Biotechnology, Siberian Federal University, 660041 Krasnoyarsk, Russia; 11 Laboratory of Population Genetics, N. I. Vavilov Institute of General Genetics, Russian Academy of Sciences, 119333 Moscow, Russia; 12 Scientific and Methodological Center, G. F. Morozov Voronezh State University of Forestry and Technologies, 394087 Voronezh, Russia

**Keywords:** laccase gene, *Populus*, *Trametes hirsuta*, transgenic aspen, 2,4,6-trichlorophenol, phytoremediation

## Abstract

**Objective:** Laccases are known to be able to degrade phenolic compounds to simpler components. The main objective of our study was to analyze this property in transgenic aspen plants carrying the laccase gene *Lac* from *Trametes hirsuta* which can be potentially used in soil phytoremediation. **Methods:** We created transgenic aspen plants carrying the laccase gene *Lac* from *Trametes hirsute* using the agrobacterial transformation of stem explants with the pBI–Lac vector containing the *Lac* gene from the white rot fungus *T. hirsuta* 072 (NCBI GenBank accession number KP027478). Transgenic plants were micropropagated and cultivated in vitro in lines. The degradation of 2,4,6-trichlorophenol (2,4,6-TCP) by plant roots was analyzed by mass-spectrometry with electron ionization using a gas chromatograph. **Results:** Although plants have their own laccases, those of fungal origin are more effective. All transgenic plants that expressed the recombinant gene degraded 2,4,6-TCP more effectively than non-transformed plants in the control (the degradation efficiency ranged 92 to 98% versus 82% in non-transformed control). Line 47Lac8 demonstrated a 16% higher efficiency than the non-transformed plants in the control. There was also an inverse relationship between the viability of a transgenic line and its level of expression of the recombinant gene. Thus, line 47Lac4 was not viable under native conditions, probably due to lignin synthesis disruptions during the initiation of secondary tissues. This is confirmed by changes in the expression of native genes of lignin biosynthesis. The rest of the transgenic lines did not differ significantly from control in wood growth and biochemistry. The transgenic plant roots were shown to preserve the ability to express the *Lac* gene ex vitro. **Conclusions:** Three transgenic lines (47Lac5, 47Lac8, and 47Lac23) with the *Lac* gene can be recommended for use in soil phytoremediation.

## 1. Introduction

Phenolic compounds are among the main pollutants in many industrial cities and areas. Unfortunately, there is no steady downward tendency in phenolic contamination of water sources and soils [1]. As shown by long-term studies around the world, chlorophenols are mainly discharged as co-products in paper industry. They are also formed in drinking water due to disinfection by chlorination, in the process of working the wood, and are also used as pesticides and herbicides. Gasses emitted by municipal incinerators contain 2,4,6-trichlorophenol (2,4,6-TCP), which then drops with precipitation [2,3]. Some harmful substances, which are brought down to the soil and water during precipitation, such as chlorophenols, polycyclic aromatic hydrocarbons, benzenes, and toluenes, are stable in the environment and have carcinogenic and mutagenic effects [4]. Phenolic compounds are stable compounds with bactericidal and fungicidal properties [5]. Chlorophenols are the result of a combination of phenol and chlorine and are usually more stable and resistant than phenol [6]. At the same time, 2,4,6-TCP is difficult to naturally decompose and has strong lipophilic properties, so it is easily absorbed by water or soil. 2,4,6-TCP is one of the most common waste products derived from various industries. At the same time, its peculiar chemical structure slows its decomposition in the environment, making it more dangerous [7]. Therefore, this study focuses on cleaning up this class of compounds from the environment using phytoremediation. The ability of fungi to degrade a wide range of harmful chemicals has incited interest in their use in bioremediation [8]. Laccases can potentially be used for the bioremediation of contaminated soil since they can oxidize toxic organic pollutants such as chlorophenols, polycyclic aromatic hydrocarbons, lignin-related structures, organophosphorus compounds, phenols, and azo dyes [4].

Fungal laccases have already been successfully used for the discoloration and detoxification of industrial waste water, as well as in wastewater treatment [9,10] with an emphasis on fungal laccases that can also be used for soil bioremediation. The use of white rot fungus (WRF) protein extracts and fungi immobilized on various substrates showed a high effectiveness in bioremediation, although the effect was short-lived [9,10]. The microbiological purification by chlorophenols, particularly of soil, does not seem to be efficient either. Therefore, phytoremediation by transgenic plants carrying WRF laccase genes was considered the most promising alternative. In early studies, transformed tobacco plants (*Nicotiana tabacum*) used as model plants for genetic transformation decreased the content of 2,4,6-TCP in the culture medium by 76% [11]. Transformed *Arabidopsis thaliana* plants expressing the *Lcc9* gene from *Laccaria bicolor* were able to discolor triphenylmethane dyes used in industrial dyeing [12]. The creation of woody perennials with a recombinant WRF laccase gene seems even more promising for phytoremediation. Although herbaceous plants can be used for phytoremediation, their use is uneconomical because of the small root system area and lower resistance to biotic and abiotic factors, thus requiring the frequent additional planting of transgenic plants, as well as specialized care and monitoring [13]. Therefore, transgenic woody plants are considered the most promising and cost-effective option. Until now, however, to our knowledge, there have been no publications describing any studies of using trees expressing recombinant fungal laccases for phytoremediation.

Plants belonging to *Populus* are widely used in transformation studies due to their physiological and ecological characteristics and their wide use in various industries [14,15,16,17]. These species are characterized by rapid growth and increase in biomass; high adaptability to various soil and water conditions; significant tolerance to air, water, and soil pollution; mass production of planting material; easy vegetative cloning; and good planting efficiency with minimal necessary care [18]. Aspens are fast-growing species of the genus *Populus* that are widespread in the northern hemisphere [19]. Due to high resistance and a wide range of growth, aspens are the most appropriate among *Populus* for genetic transformation [20]. Aspen plants have already been engineered with genes for lignin modification [21], increased productivity [22], resistance to pests [23], herbicides [24], and drought [25,26]. In Russia, aspen is the second most widely distributed broadleaf tree after birch [27]. In the field of phytoremediation research, transgenic *Populus* species have been used to remove toxic metals from soil. However, woody plants, and in particular, *Populus* species, have not been used to decompose organic compounds [28]. Considering the role of fungal laccases in the degradation of phenolic compounds, we developed transgenic aspen plants with an overexpression of the *Lac* gene from the *Trametes hirsuta* fungus. We used this fungus because laccase produced by *T. hirsuta* belongs to the group of high redox potential laccases, and it oxidizes various substrates of phenolic nature with high efficiency. In addition to high catalytic activity, this enzyme has high stability. This paper discusses the potential use of the obtained transgenic plants for degrading 2,4,6-TCP via their root system. Therefore, the aim of our research was to provide a detailed analysis of the phenotypic and biochemical characteristics of transgenic plants in order to assess whether the use of this genetic construct negatively impacts plant metabolism. In addition, we conducted a preliminary analysis of the possibility of decomposing 2,4,6-TCP by transgenic plants in a nutrient medium.

## 2. Materials and Methods

### 2.1. Plant Material and Genetic Transformation

The study used hybrid aspen plants (*Populus tremula* × *Populus tremuloides* genotype 47-1) kindly provided by the Latvian State Forest Research Institute “Silava”. This hybrid grows in a wide variety of boreal climatic conditions, has a faster growth rate compared to its parents, is resistant to pathogens, has a compact crown and fewer lateral branches, and prefers high humidity but can also grow in dry habitats. Productivity is 180–200 m^3^/ha at the age of 12 years when planting 1100 trees per 1 ha. It also has higher resistance to heart rot. The transgenic plants were generated by the agrobacterial transformation of stem explants with the pBI–Lac vector [29] containing the *Lac* gene from the white rot fungus *T. hirsuta* 072 (NCBI GenBank accession number KP027478) [30,31]. The vector construct used the constitutive promoter 35S and the nopalin synthetase terminator *nos* with a white poplar cellulose signal peptide. The kanamycin resistance gene *nptII* was used as a selectable gene. The total DNA was isolated from each aspen line and tested through PCR for the integration of the selectable gene *nptII* and the target gene *Lac.*

Transgenic plants were micropropagated and cultivated in vitro in lines on Woody Plant Medium (WPM) [32] containing vitamins MS [33] without adding hormones in glass jars with a volume of 330 mL. The incubation period lasted for four weeks with a photoperiod of 12 h of daylight with an intensity of 4000 lux and 12 h of darkness at 24 °C. After the stages of in vitro cultivation, the plants were transplanted into soil substrate. Each line was represented by 40 individual plants. Adaptation to greenhouse conditions lasted one month under conditions of high humidity (~90%) with slight shading (~2500 lux) at 25 °C. After that, the plants were allowed to grow in the greenhouse for another three months at humidity of ~60% with light of ~7000 lux at 24 °C for 15 h of daytime and 9 h of night-time. Later they were transferred to seminatural cultivation conditions (outdoor potted plants). In such conditions, the plants were allowed to grow for four years, during which they were annually transplanted into larger pots and wintered in natural conditions. The plants were set on the ground randomly in order to avoid variations due to edge effects and local conditions.

### 2.2. Confirmation of the Presence of Recombinant Gene

The plant total DNA was extracted from young leaves of aspen plants grown in greenhouse for three months using DNeasy Plant Mini Kit (QIAGEN, Venlo, The Netherlands), according to the manufacturer’s instructions. The initial screening for transgenic lines was carried out using the PCR primers for the *nptII* gene (*nos* 5′-CGCGGGTTTCTGGAGTTTAATGAGCTAAG-3′; *nptII* 5′-GCATGCGCGCCTTGAGCCTGG-3′, the amplified product size of 741 bp) and the recombinant *Lac* gene (*Lac-up* 5′-TCATTGACAACTTGACGAAC-3′; *Lac-low* 5′-GCAGGATAGCCGAGTTAATAC-3′, amplified product size of 741 bp). PCR amplification was performed in 25 μL of a mixture of genomic DNA (50 ng), primers (7 pmol each), and reagents from an Encyclo Plus PCR kit (Evrogen JSC, Moscow, Russia). The amplification parameters were as follows: hot start denaturation at 96 °C for 3 min, denaturation at 95 °C for 45 s, annealing at 62 °C in case of the *nptII* gene or 60 °C in case of the *Lac* gene for 45 s, and 1 min elongation at 72 °C; 31 cycles. Reactions were set up in a MJ MiniTM Gradient Thermal Cycler (Bio-Rad, Hercules, CA, USA).

### 2.3. Gene Expression Analysis (RT-qPCR)

Plant total RNA was extracted from young leaves of all seven-month-old transgenic and control lines growing in the seminatural conditions using the Extract RNA kit (Invitrogen, Waltham, MA, USA), as described in the manufacturer’s protocol (http://www.invitrogen.com, accessed on 23 October 2024). Root total RNA was isolated from ex vitro-grown four-year-old plants. Only tips of young roots no longer than 1.5 cm were used. The root RNA was isolated according to the protocol proposed in [34].

For cDNA synthesis, 500 ng of total RNA were used. RNA concentration was measured using a microvolume spectrophotometer Nanodrop 2000 (Thermo Fisher Scientific, Waltham, MA, USA). The removal of DNA residues was performed using the TURBO DNA-free Kit (Thermo Fisher Scientific, Waltham, MA, USA). The synthesis of cDNA was carried out using M-MuLV reverse transcriptase RNase H (SibEnzyme, Novosibirsk, Russia) and oligo-(dT)18 primer (Syntol, Moscow, Russia) at 37 °C for 60 min.

The levels of gene expression were assessed by real-time quantitative PCR (RT-qPCR) using cDNA preparations. The RT-qPCR method and data analysis are described in detail in [35]. Actin (*Act*) and ubiquitin (*UBQ*) genes were used as reference genes in the gene expression analysis. The primers used in RT-qPCR are listed in Table 1. The expression level of genes associated with lignin biosynthesis was studied to analyze changes in lignin composition [36].

### 2.4. Quantitative Western Blot Analysis and Determination of Total Protein Concentration in Extracts

Total protein was isolated from greenhouse-grown 6-month-old transgenic and control plants using an extraction buffer consisting of 0.175 MTris/HCl (pH 8.8), 5% SDS, 15% glycerol, and 0.3 M mercaptoethanol [37]. The electrophoretic separation of proteins was performed in 12% polyacrylamide gel, according to the standard protocol presented in [38]. A mixture of proteins with known molecular weights of 20, 26, 34, 50, 90, and 120 kDa were used as markers (Thermo Fisher Scientific, Waltham, MA, USA). Equal amounts of plant total protein (120 μg) were loaded in each lane. The total protein concentration was measured by spectrophotometry using the Shimadzu UV-1800 spectrophotometer (Shimadzu, Kyoto, Japan) at a wavelength of 562 nm and a Pierce BCA Protein Assay kit (Thermo Fisher Scientific, Waltham, MA, USA). As positive control, we used a homogenous preparation of laccase from *T. hirsuta* 072 obtained at the Institute of Biochemistry of the Russian Academy of Sciences (currently part of the Federal Research Centre “Fundamentals of Biotechnology” of the Russian Academy of Sciences, Moscow, Russia). The separated polypeptides were electrically transferred onto nitrocellulose membrane (Bio-Rad, Feldkirchen, Germany) by semi-dry transfer on a TE 70 PWR transblotter (Amersham Biosciences, Piscataway, NJ, USA). Primary rabbit anti-laccases were obtained at the Institute of Cell Biophysics of the Russian Academy of Sciences (Pushchino, Russia). Hybridization with primary antibodies was carried out at 8 °C for 18 h. Hybridization with secondary antibodies was conducted at room temperature for 2 h. The secondary antibodies were monoclonal goat anti-rabbit antibodies conjugated with alkaline phosphatase (Sigma-Aldrich, St. Louis, MO, USA). Immune complexes were detected using a BCIP/NBT ready-to-use substrate (Serva, Heidelberg, Germany). For higher-quality imaging in Western blot analysis, a Pierce TM Western Blot Signal Enhancer (Thermo Fisher Scientific, Waltham, MA, USA) was used.

### 2.5. Native Polyacrylamide Gel Electrophoresis (PAGE) and Zymogram Analysis

The activity of recombinant laccase from *T. hirsuta* in the aspen plants was assessed by zymography. For this purpose, we used preparations of total protein extracts from greenhouse-grown 6-month-old transgenic and control plants. The samples were run in 12% native-PAGE gel. Each well was loaded with 120 μg of plant total protein. As positive control, we used a purified laccase preparation from *T. hirsuta* with a concentration of 4.4 mg/mL and specific activity of 49.8 U/mg (ε436 = 29300 M^−1^cm^−1^) with ABTS as substrate [39]. Laccase, 0.15 μg/well, was loaded. The gel was incubated at 30 °C for 1 h in 0.1 M sodium acetate buffer solution (pH 4.0) containing 10-2M ABTS (2,2′-azino-bis(3-ethylbenzothiazoline-6-sulfonic acid).

### 2.6. Growth Indicators

Forty plants per line were included in the study. The height, stem diameter, trunk volume, and the number of branches were measured at the age of four years for plants growing in semi-natural conditions. The stem length was measured from the root neck to the apical bud. The stem diameter was measured at the base of the root neck. The trunk volume was measured according to the formula proposed by Shani [40]. The leaf area was calculated in the second year of vegetation using LAMINA software [41].

### 2.7. Determination of Karyotype

For each preparation, two to four 0.5–2 cm long roots were cut off from the obtained in vitro rooted aspen plants. In order to obtain prometaphase chromosomes and accumulate mitoses, the roots were placed in a test tube with a water solution of 9-aminoacridine (9-AMA) DNA intercalator, 1 μg/mL, and kept at 0 °C for 24 h [42,43]. The roots were then transferred to Carnoy’s fixative solution (ethanol/glacial acetic acid, 3:1) and placed at +4 °C. The fixative solution was renewed once more after 24 h. Next, the samples were stained with 1% acetocarmine solution in 45% acetic acid at room temperature for 20 min. The obtained preparations were transferred to 96% ethanol for dehydration and stored at 4 °C. The chromosome preparations were dried and stained with A-T specific fluorescent dye DAPI (4′, 6-diamidino-2-phenylindole). The finished preparations were examined using an Olympus BX43 fluorescence microscope (Olympus Corporation, Tokyo, Japan) equipped with a CCD 3501 camera (CINOGY Technologies GmbH, Göttingen, Germany). Fifteen metaphase plates per line were examined, and a chromosome count was performed using the AutoKario 1.1 chromosome analysis program, a macro to the ImageJ software v. 1.53t and 1.51s (https://soft.mydiv.net/win/download-ImageJ.html, http://www.bioinformatix.ru/plaginyi-i-makrosyi-programmyi-imagej/makros-k-programme-imagej-autokario-1.1-avtomaticheskoe-kariotipirovanie-po-sravneniyu-gistogramm.html, accessed on 23 October 2024).

### 2.8. Analysis of Main Wood Components

The contents of lignin, cellulose, and pentosans (hemicellulose) were measured in six plant lines (47Lac5, 47Lac7, 47Lac8, 47Lac11, 47Lac23, and 47-1). Samples were taken from the base of the root neck of 10 four-year-old plants per line. The total lignin content was measured as proposed by Foster [44]. The cellulose content was measured using the Kurschner–Hanak nitrogen–alcohol method [45]. The specific content of pentosans in wood was estimated by the modified Tollens method [46,47].

### 2.9. Analysis of Phenolic Degradation

The potential for phenolic degradation was studied in an in vitro experiment on 2,4,6-TCP degradation by the roots of transgenic plants. Four most effective transgenic lines (47Lac4, 47Lac5, 47Lac8, and 47Lac23) and the control line 47-1 were used. Twenty plants per line were grown in bioreactors with liquid WPM for three weeks to form a developed root system. Each line was presented in three replicates. Next, 2,4,6-TCP was added to the nutrient medium to a concentration of 10 µM (this sublethal dose was determined experimentally). After 2 weeks of incubation, the culture medium was sampled to analyze changes in the 2,4,6-TCP concentration. The latter was measured by mass spectrometry with electron ionization using a gas chromatograph TRACE 1310 GC with mass spectrometric detector TSQ 9000 and autosampler AI/AS 1310 (Thermo Fisher Scientific, Waltham, MA, USA), as well as a chromatographic column, TR-5MS GC 30 m × 0.25 mm ID, 0.25 µm (Thermo Fisher Scientific, Waltham, MA, USA). For reliable analysis, samples of culture medium were taken at three time points: before adding 2,4,6-TCP, immediately after its addition, and after two weeks of plant incubation with 2,4,6-TCP, respectively. All samples were analyzed in triplicate. The chromatographic data were processed using the Chromeleon v. 7.2 software (https://www.thermofisher.com/order/catalog/product/CHROMELEON7, accessed on 17 August 2024).

### 2.10. Statistical Data Analysis

The principal component analysis (PCA), descriptive statistics, analysis of variance, and construction of the box plots were performed with the Statistica v. 10.0 software (https://statistica.software.informer.com/10.0, accessed on 17 August 2024) and Microsoft Excel 2010 (v. 14.0.7268.5000).

## 3. Results

### 3.1. Confirmation of Transgenic Status

**Figure 1:**
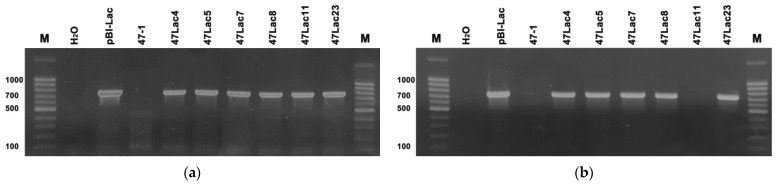
PCR analysis of *nptII* (**a**) and *Lac* (**b**) gene expression (the expected amplicon sizes are 690 and 741 bp, respectively). M—a standard molecular marker of 1 Kbp (SibEnzyme, Novosibirsk, Russia); H_2_O—negative PCR control; pBI-Lac—plasmid DNA (positive control); 47-1—a non-transgenic control line; 47Lac11—transgenic control line.

Agrobacterial transformation was used to generate six transgenic lines (47Lac4, 47Lac5, 47Lac7, 47Lac8, 47Lac11, and 47Lac23). Line 47Lac11 was selected as transformation control: it participated in the transformation process but without the target gene *Lac*. DNA was isolated from plants of all lines and analyzed to confirm their transgenic status (Figure 1). The selectable kanamycin resistance gene *nptII* was found in all transgenic lines, including 47Lac11 (Figure 1a). The DNA analysis showed the presence of the target gene *Lac* in lines 47Lac4, 47Lac5, 47Lac7, 47Lac8, and 47Lac23 (Figure 1b).

**Figure 2:**
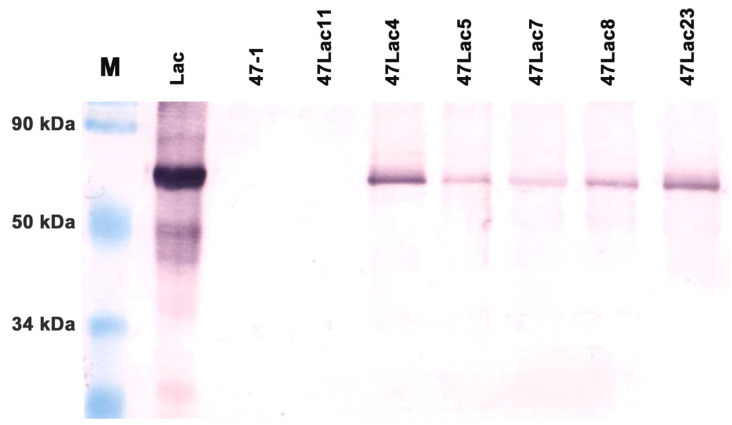
Western blot analysis of protein extracts of transgenic aspens carrying the recombinant *Lac* gene. M—standard protein molecular marker; Lac—fungal extract; 47-1—non-transgenic control; 47Lac11—transgenic negative control; 47Lac4, 47Lac5, 47Lac7, 47Lac8, and 47Lac23 are transgenic lines.

Western blotting confirmed the presence of the recombinant protein Lac of an appropriate size (67 kDa) [31] in all five selected transgenic aspen lines carrying the *Lac* gene. The recombinant protein was detected stably in all replicates of the analysis (Figure 2).

**Figure 3:**
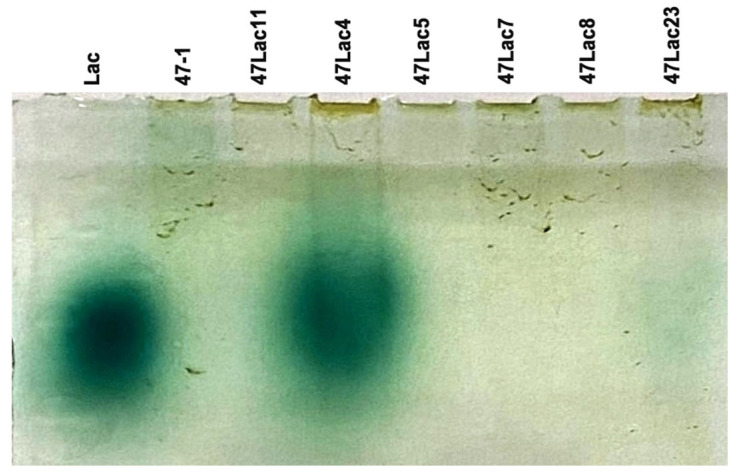
Zymogram analysis of protein extracts of transgenic aspens carrying the recombinant *Lac* gene. M—standard protein molecular marker; Lac—fungal extract; 47-1—non-transgenic control; 47Lac11—transgenic negative control; 47Lac4, 47Lac5, 47Lac7, 47Lac8, and 47Lac23 are transgenic lines.

As shown by the zymogram, the highest activity of the Lac protein was observed in line 47Lac4 (Figure 3). Line 47Lac23 also demonstrated high activity of the recombinant protein. The rest of transgenic lines had lower activity.

**Figure 4:**
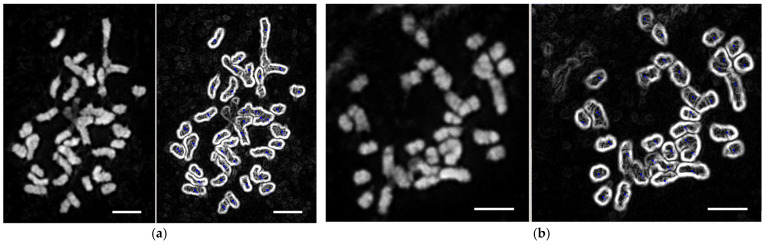
Micrographs of metaphase plates for counting chromosomes of aspen plant lines 47-1 (**a**) and 47Lac23 (**b**); bars represent scale of 4 μm.

The karyotype analysis of the hybrid aspen line 47-1 and its transformants determined the limits of chromosome number variability; the modal number was 38. The chromosomes of the studied species are small, with a bi-armed morphology, but there are chromosomes with an unidentified position of the centromere. The obtained results suggest that all studied lines are diploids (2n = 38) with a base number x = 19 (Figure 4).

### 3.2. Gene Expression of the Transgenic Gene Lac and the Native Genes of Lignin Biosynthesis

**Figure 5:**
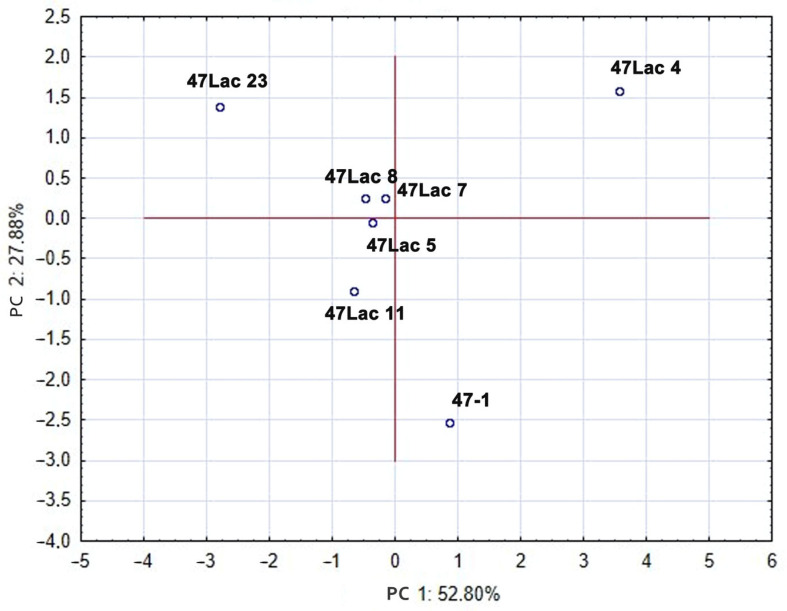
PCA of expression levels of lignin biosynthesis gene; 47-1—non-transgenic control; 47Lac11—transgenic negative control; 47Lac4, 47Lac5, 47Lac7, 47Lac8, and 47Lac23 are transgenic lines.

The expression of the *Lac* gene and the native genes of lignin biosynthesis was studied on RNA samples from leaves of 7-month-old aspen plants when the lignification process is the most intense. The RT-qPCR analysis of plant cDNA isolated from transgenic aspen lines showed the expression of the recombinant *Lac* gene in all samples except the control lines 47-1 and 47Lac11. The highest expression of the recombinant *Lac* gene was found in line 47Lac4. The level of relative expression of this gene in genotype 47Lac4 was considered a reference value and equaled 1 for comparison with other lines. The next highest level of the recombinant gene expression relative to the reference value was 0.346 in line 47Lac23; the lowest relative expression was 0.013 in line 47Lac7. For lignin biosynthesis genes, the level of relative expression in control line 47-1 was taken as equal to one. Changes in the expression of these genes were observed in all transgenic lines (Table 2). As shown by the gene expression results, transgenic lines 47Lac5, 47Lac7, and 47Lac8 were the most similar in expression levels and formed one group. Plants of the control lines, 47-1 and 47Lac11, showed expression levels quite similar to each other but different from those of transgenic plants with the *Lac* gene. The *Lac*-gene expression in lines 47Lac4 and 47Lac23 differed greatly from those in the other lines (Figure 5).

The analysis of *Lac* gene expression in roots showed lower expression levels than in plant leaves (Table 3). However, the noted inter-line variation in the recombinant gene expression persisted. The highest expression was also demonstrated by line 47Lac23, followed by 47Lac8, and 47Lac5, while the lowest was for 47Lac7.

### 3.3. Plant Biometry Data

Following in vitro rooting, the plants adapted to greenhouse conditions. Then, they were transplanted into large diameter pots and transferred to semi-natural cultivation conditions. Not all plants, however, were able to normally adapt and grow in greenhouse conditions. All plants of line 47Lac4 (characterized with the highest *Lac* gene expression) had difficulty adapting to greenhouse conditions and subsequently died within one year. The plants of all lines, except 47Lac4, were grown in semi-natural conditions under the influence of all biotic and abiotic factors for four years. At the end of the fourth year of vegetation, the height, diameter, and volume of all plants of all the lines were measured (Table 4). There were no significant differences in trunk growth parameters: all of these were similar in the transgenic plants and controls. However, the leaf area and the number of branches in the transgenic lines differed from the controls. Line 47Lac23 had a smaller leaf area and a greater number of branches. Line 47Lac5 was characterized by a decreased number of branches, while line 47Lac8 showed a larger leaf area.

### 3.4. Content of Pentosans (Hemicellulose), Cellulose, and Total Lignin

The results of analysis of such main components of wood as pentosans (hemicellulose), cellulose, and total lignin are presented in Table 5. No statistically significant differences were found. However, the transgenic lines carrying the *Lac* gene showed slightly decreased pentosans and increased cellulose content. The lignin content was approximately the same in all lines.

### 3.5. Phenolic Degradation Data

The quantitative determination of residual 2,4,6-TCP in the nutrient medium was performed for transgenic lines with the confirmed high expression of the *Lac* gene (47Lac4, 47Lac5, 47Lac8, and 47Lac23) and the non-transgenic control (47-1). Line 47Lac7 was excluded from the study because of an extremely low expression level in the roots of the transgenic plants. All studied lines showed the ability to degrade phenolic compounds (Table 6). All transgenic lines showed higher levels of 2,4,6-TCP degradation than the control line 47-1. The largest and most statistically significant difference from the control was noted for line 47Lac8. It degraded up to 98% of 2,4,6-TCP, which was 16% of the 2,4,6-TCP amount in control (Table 6). Although we found no direct relationship between *Lac* gene expression and 2,4,6-TCP degradation efficiency, line 47Lac8 had fairly high expression levels for the recombinant *Lac* gene.

## 4. Discussion

The rapid growth of cities and industries in recent decades has led to serious pollution of water bodies and soils. Therefore, a wide range of strategies are currently being developed to mitigate the effects of global pollution. However, this proved to be a difficult task because of the great variety and amounts of contaminants [48]. One of biotechnological strategies for the detoxification of harmful contaminants makes use of laccase enzymes. Laccases, which belong to the family of multicopper oxidases, are considered universal enzymes due to their low substrate specificity and are capable of oxidizing a large number of phenolic and non-phenolic molecules [4,49]. Laccases use oxygen as the electron acceptor and generate water as byproduct [50]. These enzymes—diverse in specificity and activity—are common in fungi, plants, bacteria, lichens, and insects [51]. The remediating potential of fungal laccases is of great interest to researchers. Most of these laccases have a high redox potential (E°) and can oxidize substrates with high E°. Therefore, fungal laccases are successfully used for bioremediation through the oxidation of polycyclic aromatic hydrocarbons, dyes, plastics, active pharmaceutical ingredients, and phenolic compounds. It should be noted that laccases from white rot fungi (*Trametes*) have a potential to degrade phenolic compounds [48]. *Trametes versicolor* was successfully used to degrade and eliminate phenolic contaminants [52]. Protein extracts from *T. pubescens* demonstrated the ability to degrade various chlorophenols [53]. Free purified laccase from *T. villosa* was successfully used to degrade bisphenol A [54]. Laccase isolated from *T. trogii* can degrade and discolor synthetic dyes [55]. An extract from the white rot fungus *T. hirsuta* was shown to degrade chloramphenicol [56]. To obtain transgenic plants with potential for the phytoremediation of phenolic compounds, we chose laccase from *Trametes hirsuta* strain 072 [30,31]. This laccase has a very high redox potential of 790 mV, a high catalytic efficiency compared to other fungal laccases, and a broad substrate specificity [30]. It was due to the above parameters that the sequence of the *Lac* enzyme was chosen for plant transformation.

To study the potential for the use of transgenic woody plants carrying the recombinant laccase gene for phytoremediation, we created six hybrid aspen lines based on wild-type *P. tremula* × *P. tremuloides* hybrid (genotype 47-1). The expression of the recombinant construct and protein was confirmed for five of the six obtained lines. Expression of the gene and protein was not confirmed for line 47Lac11; this line was used as transformation control because of the confirmed presence of selectable kanamycin resistance gene, *nptII*. The expression of the recombinant protein correlated with the expression level of the recombinant *Lac* gene. The genetic and phenotypic effects of hybridization depend on the preservation of, or change in, plant ploidy [57]. The genetic transformation and microclonal propagation of a plant are often accompanied by the structural rearrangements of the karyotype. A karyological study of the plants was conducted to exclude the possible emergence of new phenotypic traits due to changes in the ploidy. The study found no rearrangement in the karyotype of the control and transgenic lines.

In the plant body, laccases are more involved in the processes of lignin biosynthesis and polymerization and less in stress response [48]. Therefore, a great deal of researchers’ attention was concentrated on changes in lignin content in transgenic plants. The transgenic herbaceous plants of *Oryza sativa* [58], *N. tabacum* [11,59], and *A. thaliana* [60] were shown to have a significantly lower contents of lignin and its components compared to respective wild-type plants. No such studies of woody plants are available. This is probably due to more complex lignification processes during the secondary growth of woody plants. According to our studies, the expression of genes involved in lignin biosynthesis significantly changes in transgenic plants (Table 2). Based on the expression levels of the native lignin biosynthesis genes and the recombinant gene *Lac*, all studied lines can be divided into five groups: (1) control line 47-1, (2) transformation control 47Lac11, (3) line 47Lac4, (4) line 47Lac23, and (5) the rest of the lines. Line 47Lac11, the transformation control, showed changes in the expression of native genes, i.e., the act of transformation also affected the genotype of plant as a whole. However, there were no associated quantitative changes in the content of total lignin or other wood components in the transgenic lines (Table 5), perhaps due to the involvement of compensatory mechanisms in the biosynthesis of lignin and other wood components. However, with the very high level of *Lac*-gene expression noted in line 47Lac4, we observed a significant change in the expression of lignin biosynthesis genes, which probably disrupted normal lignification processes and resulted in the plants’ death at the age of 8 months. Generally, no detrimental effect was noted on the phenotype of the generated transgenic lines. Line 47Lac23 showed the highest level of recombinant laccase expression ex vitro and had a significantly smaller leaf blade area (Table 3 and Table 4). This could be due to a very strong change in lignification. However, just like with the recombinant *sp-Xeg* gene, such a decrease in the leaf blade area was compensated by a greater number of branches, and, hence, a greater number of leaves [61].

The increasing release of various chlorophenolic compounds into the environment presents a serious pollution problem. One of the most hazardous pollutants in this group is 2,4,6-TCP [62]. The chemical is widely used as an antiseptic, in the production of glues, in organic solvents, and in the synthesis of various agricultural chemicals [63]. 2,4,6-TCP is classified as an extremely mutagenic and carcinogenic compound [64]. 2,4,6-TCP is difficult to degrade, so any waste containing the chemical remains highly toxic for a long time [65]. The use of laccases is considered to be one of the ways of eliminating 2,4,6-TCP in the environment. Degrading 2,4,6-TCP by bacterial [66] or fungal [53] laccases is most promising, with a degradation efficiency of 90 to 100%. This approach, however, has some limitations. Therefore, the creation of transgenic plants with an overexpression of laccases is considered a promising solution [48,67]. Before performing the degradation study, we determined the sublethal dose of 2,4,6-TCP for the hybrid under study. It was found to be 10 µM, which was comparable with similar phytoremediation studies for transgenic *A. thaliana* carrying a recombinant plant laccase gene [67]. Lines 47Lac5, 47Lac8, and 47Lac23 were selected as the most appropriate candidates to degrade phenolic compounds. Our generated transgenic plants effectively degraded 92% to 98% of TCP. Such levels of degradation efficiency are very high compared to the average 70% demonstrated by herbaceous plants [11,67]. The roots of the transgenic plants were shown to express the *Lac* gene ex vitro. Hence, these lines can potentially be used for soil phytoremediation. The control line 47-1 was also able to degrade TCP quite effectively—up to 82%. Similar results were obtained for *A. thaliana* transformed with the *Lac1* gene from *Gossypium arboretum*: the degree of 2,4,6-TCP degradation by the transgenic plants in the culture medium was 70%, with a small difference (<5%) from the wild-type plants [61]. A similarly high degradation efficiency (80%) was shown by a plant laccase from *Monilinia fructigena* expressed by *Pichia pastoris* [68]. This suggests that the detoxification potential of a plant depends on a whole range of secreted compounds. Plants have their own laccases and other detoxifying substances. For example, even the *A. thaliana* genome was shown to have 15 genes very similar to those of laccases [69]. Due to secondary growth in woody plants, one may assume that they have more laccase genes. The expression dynamics of plant laccase genes and proteins with similar activities in the aspen hybrid under study need further research.

Secretory laccases are responsible not only for phenolic metabolism within the plant, but some of them help the plant detoxify its immediate soil environment [48]. This environmental function can be used to develop a novel phytoremediation system based on the ex planta conversion of phenolic pollutants. In the case of the detoxification of immediate soil by the root system, the plant cells are not directly exposed to the pollutant, which provides a clear advantage over phytoremediation strategies based on the uptake and the accumulation of pollutants by plants [67]. The use of recombinant WRF laccases—with their wider substrate specificity and higher activity—can further intensify detoxification and, therefore, make phytoremediation more effective.

## 5. Conclusions

For the first time, woody transgenic plants with the ability to decompose chlorophenolic compounds have been obtained. Among five transgenic aspen lines of a hybrid *P. tremula* × *P. tremuloides* generated in this study carrying the *Lac* gene from a strain of *Trametes hirsute*, three lines (47Lac5, 47Lac8, and 47Lac23) were able to phytoremediate the environment. The introduction of the recombinant construct was shown to increase the 2,4,6-TCP degradation efficiency by up to 92–98%, without changing the habitus and the biochemical parameters of wood. The non-transgenic control was also able to degrade 2,4,6-TCP but with about 16% lower efficiency than the transgenic plants. The expression dynamics of the plant genes enabling the degradation of phenolic compounds should be studied in future research. The preservation of the *Lac* gene expression in the roots of transgenic plants was shown ex vitro. Therefore, the created transgenic clones can be recommended for the potential phytoremediation of soil. The presented study on the potential of transgenic aspen trees for phenolic degradation is only preliminary and has been performed in vitro only. Authors certainly realize that additional experiments should be conducted in field conditions ex vitro to better understand the potential of these transgenic plants and their compounds. We are planning ex vitro experiments, but they would not be possible without the preliminary confirmation of the effectiveness of the obtained transgenes in vitro demonstrated here in our study.

## Figures and Tables

**Table 1:** Genes and PCR primers used in RT-qPCR.

Gene	NCBI GenBank Accession Number	Primer Nucleotide Sequence (5′-3′)	
		Forward	Reverse
*Act*	MN196665.1	TATGCCCTCCCACATGCCAT	CATCTGCTGGAAGGTGCTGA
*UBQ*	PQ155116.1	GTTGATTTTTGCTGGGAAGC	GATCTTGGCCTTCACGTTGT
*LacTh*	KP027478	ATTCTCAGTGGTGCCCACAGACGG	ATGGACGCGTTCGACGGAAG
*CAD6*	KF145199.1	GGTAGGAAGCAAAGTTGAAAAGTTC	TAGCCTCCGTACGTGGTGGTTCCAT
*CCoAOMT1*	KX227458.1	GTACCTCACATACTTCCTCATTGGT	TGGAAGTTTTGATTTCATCTTTG
*CCR1*	MW928493.1	AGTTTTCCATACTGCTTCTGTC	ACAACATCAGGGCTCCTATTGGGGT
*Pxp 3-4*	X97351.1	CAGCTTACTCCAACATTTTATGACC	GTATTGTCCAACAAAAGTGAACCA
*4CL*	MK256749.1	CTTTGTTAATAGCCCATCCAG	TGATTTCACAGCAAATGCAAC
*MYB152*	PQ178447.1	TCGTATCTGAACTGGACCAAAATAG	AGGGACTAAGATTTCATGGGGTTC

**Table 2:** RT-qPCR analysis of expression of the transgenic gene *Lac* and the native genes of lignin biosynthesis in seven-month-old transgenic and control aspen lines.

Line	*Lac*	*CAD6*	*CCoAOMT1*	*CCR1*	*Pxp3-4*	*4CL*	*MYB152*
47-1 (control)	-	1	1	1	1	1	1
47Lac4	1	0.44	0.89	1.26	2.53	1.35	1.65
47Lac5	0.031	0.52	0.75	0.66	1.54	0.74	1.39
47Lac7	0.0013	0.48	0.75	0.8	1.26	0.68	2.26
47Lac8	0.159	0.37	0.85	0.51	1.49	0.8	1.15
47Lac11 (control)	-	0.59	0.78	0.7	0.95	0.84	0.96
47Lac23	0.346	0.26	0.3	0.38	0.75	0.47	0.94

**Table 3:** RT-qPCR analysis of the expression of transgenic *Lac* gene in roots of four-year-old transgenic and control aspen lines.

Line	*Lac* Relative Expression Level ± Stand. Error
47-1 (control)	-
47Lac5	3.48 ± 0.13
47Lac7	1.00 ± 0.03
47Lac8	32.11 ± 2.34
47Lac23	45.89 ± 4.55

**Table 4:** Growth rate indicators, number of branches, and leaf areas (±SD) in 4-year-old aspens of transgenic and control lines in semi-natural conditions.

Line	Height, cm	Stem Diameter, mm	Volume, m^3^	Number of Branches	Leaf Area, mm^2^
47-1 (control)	288.6 ± 8.61	19.53 ± 0.57	0.110	34.3 ± 1.73	5716.0 ± 182
47Lac5	285.3 ± 10.43	18.68 ± 0.78	0.100	29.1 ± 0.98 *	6028.9 ± 170
47Lac7	268.7 ± 9.35	18.88 ± 0.79	0.096	36.8 ± 1.77	6585.2 ± 153
47Lac8	294.0 ± 9.71	21.01 ± 10.01	0.130	30.9 ± 1.3	6661.8 ± 171 *
47Lac11 (control)	279.0 ± 12.6	20.33 ± 0.95	0.115	37.0 ± 1.44	5718.4 ± 165
47Lac23	277.8 ± 11.53	19.23 ± 0.75	0.103	38.6 ± 1.2 *	3653.5 ± 155 *

* significantly different from 47 to 1 at *p* ≤ 0.05 based on ANOVA; 40 plants were measured per line.

**Table 5:** Percentages of pentosans (hemicellulose), cellulose, and total lignin (±SD) in 4-year-old aspens of transgenic and control lines.

Line	Pentosans (Hemicellulose)	Cellulose	Lignin
47-1 (control)	19.0 ± 0.99	41.8 ± 1.56	30.3 ± 1.50
47Lac5	16.9 ± 0.78	42.1 ± 1.67	29.9 ± 1.59
47Lac7	17.8 ± 0.63	42.3 ± 1.88	28.9 ± 1.21
47Lac8	18.0 ± 0.73	42.8 ± 1.70	29.2 ± 1.33
47Lac11 (control)	18.6 ± 0.87	41.3 ± 1.95	29.4 ± 1.40
47Lac23	17.8 ± 0.85	43.2 ± 1.73	30.1 ± 1.49

**Table 6:** Residual 2,4,6-TCP (after 2-week incubation in bioreactor with 10 µM 2,4,6-TCP).

Line	Residual 2,4,6-TCP, µM
47-1 (control)	1.793 ± 0.874
47Lac4	0.363 ± 0.030
47Lac5	0.414 ± 0.035
47Lac8	0.199 ± 0.037
47Lac23	0.783 ± 0.074

## Data Availability

Data are contained within the article and available from the authors upon request.
